# CASPR2 antibody associated neurological syndromes in children

**DOI:** 10.1038/s41598-023-28268-x

**Published:** 2023-02-06

**Authors:** Liwen Wu, Fang Cai, Zhihong Zhuo, Dejun Wu, Tianyi Zhang, Haiyang Yang, Hongjun Fang, Zhenghui Xiao

**Affiliations:** 1 Neurology department, Hunan Children’s Hospital, The School of Pediatrics, Hengyang Medical School, University of South China, Changsha, Hunan China; 2 Neurology department, Chenzhou Children’s Hospital, Chengzhou, Hunan China; 3 Pediatric Department, The First Affiliated Hospital of Zhengzhou University, Zhengzhou, Henan China; 4 Longshan People’s Hospital, Xiangxi Autonomous Prefecture, Hunan China; 5 Yiyang Central Hospital, Yiyang, Hunan China; 6 Hunan Key Laboratory of Pediatric Emergency Medicine, Hunan Children’s Hospital, The School of Pediatrics, Hengyang Medical School, University of South China, Changsha, Hunan China

**Keywords:** Immunology, Neuroscience, Diseases, Neurology

## Abstract

To strengthen the understanding of the clinical features for CASPR2 neurological autoimmunity in children. A multicenter retrospective and prospective analysis of CASPR2 autoimmunity was conducted. Twenty-six patients were enrolled, including 25 with serum positivity and 3 with cerebrospinal fluid (CSF) positivity; 5 patients were co-positive with anti-NMDAR or anti-GABABR antibodies. Eleven patients (who manifested with refractory epilepsy, psychobehavioral abnormalities or germinoma) presented with low antibody titers, relatively normal MRI/EEG/CSF examinations, and poor response to immunotherapy and were thus considered false positive (42.3%). Fifteen patients were diagnosed with autoimmune encephalitis/ encephalopathy/ cerebellitis (including 1 whose condition was secondary to Japanese encephalitis). The most common symptoms included disorders of consciousness (10/15), fever (8/15), psychological symptoms/abnormal behaviors (8/15), sleep disorders (8/15), seizures (7/15), movement disorders (5/15), autonomic symptoms (5/15). Brain MRI revealed abnormalities in 10 patients (66.7%). Electroencephalography (EEG) recordings revealed a slow wave background in 13 patients (86.7%). Five patients showed elevated WBCs in CSF, and 4 patients showed elevated protein levels in the CSF. Thirteen patients received immunotherapy (rituximab was adopted in 2 cases) and recovered well. Two patients received symptomatic treatment, and the recovery was slow and accompanied by emotional abnormalities and developmental delay. Autoimmune encephalitis is the most common clinical phenotype; it can be secondary to Japanese encephalitis. Rituximab can be used in patients who respond poorly to conventional immunotherapy. The high false-positive rate of anti-CASPR2 in refractory epilepsy and the psychobehavioral abnormalities needs to be explored further.

## Introduction

Contactin-associated protein-like 2 (CASPR2) is a transmembrane cell adhesion protein of the neurexin family that is expressed in neurons of the central and peripheral nervous systems^1^. It plays a role in the localization of voltage-gated potassium channel (VGKC) complex, CASPR2 can promote the aggregation and development of K channels (Kv1.1 and Kv1.2) in the proximal nodal region, which helps to form axons and maintain the stability of axons^2^. CASPR2 antibody targets multiple epitopes of CASPR2 protein, which may lead to brain and peripheral neuropathy by destroying axon potassium current^3^. CASPR2 autoantibody disease has a variable clinical phenotype that includes cerebellar syndromes, epilepsy, pain syndromes, movement disorders, psychosis, and associations with neoplasms such as thymoma. Middle-aged and elderly men are the most commonly afflicted by this condition^4^. Qin et al. reported 25 patients, the median age of symptom onset was 42 years old, 8 patients met the criteria for limbic encephalitis, epileptic seizure occurred in 6 of these 8 patients; 4 patients were diagnosed as Morvan syndrome^5^. However, data on CASPR2 autoimmunity in children are very limited. The Mayo laboratory reported 6 children with CASPR2 antibodies, encephalopathy, neuropsychiatric symptoms, and seizures were common, a neuropathic pain syndrome was the most common finding^6^. Therefore, the phenotypes, treatment strategies and outcomes of CASPR2 autoimmunity in children have great heterogeneity. Clinicians must continue to explore and summarize the incidence, clinical features, phenotypes, auxiliary examinations, diagnosis, treatment strategies, and prognosis of CASPR2 autoimmunity in children, for in-depth study and understanding.

## Methods

### Patient subjects and ascertainment

Patients under 18 years of age with clinically suspected neurological autoimmunity were identified through neural autoantibody evaluation at Hunan Children’s Hospital, Chenzhou Children's Hospital, Longshan People's Hospital, the First Affiliated Hospital of Zhengzhou University and Yiyang Central Hospital. Antibodies against NMDAR-IgG, AMPA1-IgG, AMPA2-IgG, LGI1-IgG, CASPR2-IgG, GAD65-IgG, GABABR-IgG, MOG-IgG, GFAP-IgG and AQP4-IgG were measured in serum and/or cerebrospinal fluid (CSF). Clinical data were recorded, and patients were followed up accordingly. The main collected clinical data included: demographic features, neurological symptoms and signs, laboratory tests, imaging results, treatment strategies, efficacy and prognosis evaluation, the follow-up information. Written informed consent was obtained from the patients and his parents. The study was approved by the Ethics Committees of Hunan Children's Hospital and four other centers. All methods were performed in accordance with the relevant guidelines and regulations.

Since 2020, we have performed retrospective analyses and prospective observations for CASPR2 neurological autoimmunity in multiple centers. The time span of observation was from October 2014 to October 2021.The inclusion criteria: all the enrolled patients were highly clinically suspected of having immune encephalitis, encephalopathy, or epilepsy of unknown cause with Epilepsy and Encephalopathy (APE2) score ≥ 4^9^; and the patients with positive CASPR2 antibodies in serum and/or CSF. The exclusion criteria: without neurological symptoms, or negative of CASPR2 antibodies, or with incomplete data or loss of follow-up. Eventually, there were 12 patients enrolled retrospectively, and 14 patients enrolled prospectively.

### Autoantibody testing

Specimens were tested for NMDAR-IgG, AMPA1-IgG, AMPA2-IgG, LGI1-IgG, CASPR2-IgG, GAD65-IgG, GABABR-IgG, MOG-IgG, GFAP-IgG and AQP4-IgG using a cell-based assay (CBA). The CSF was not diluted, while the serum was diluted with 1:10. HEK293T cells were cotransfected with the target antigen and pcDNA3.1-EGFP. Thirty-six hours after transfection, the HEK293T cells were fixed with 4% paraformaldehyde for 20 min. GFAP-IgG detection required additional permeabilization with 0.1% Triton X-100 in phosphate-buffered saline (PBS) for 20 min. Cells were incubated with the specimens for 2 h and then immunolabeled with an AlexaFluor 546 secondary antibody against human IgG (1:1000; Thermo Scientific) for 1 h at room temperature. Images were acquired using a Zeiss Axiovert A1 fluorescence microscope. One hundred healthy control serum specimens were provided by normal volunteer donors.

**Figure 1:**
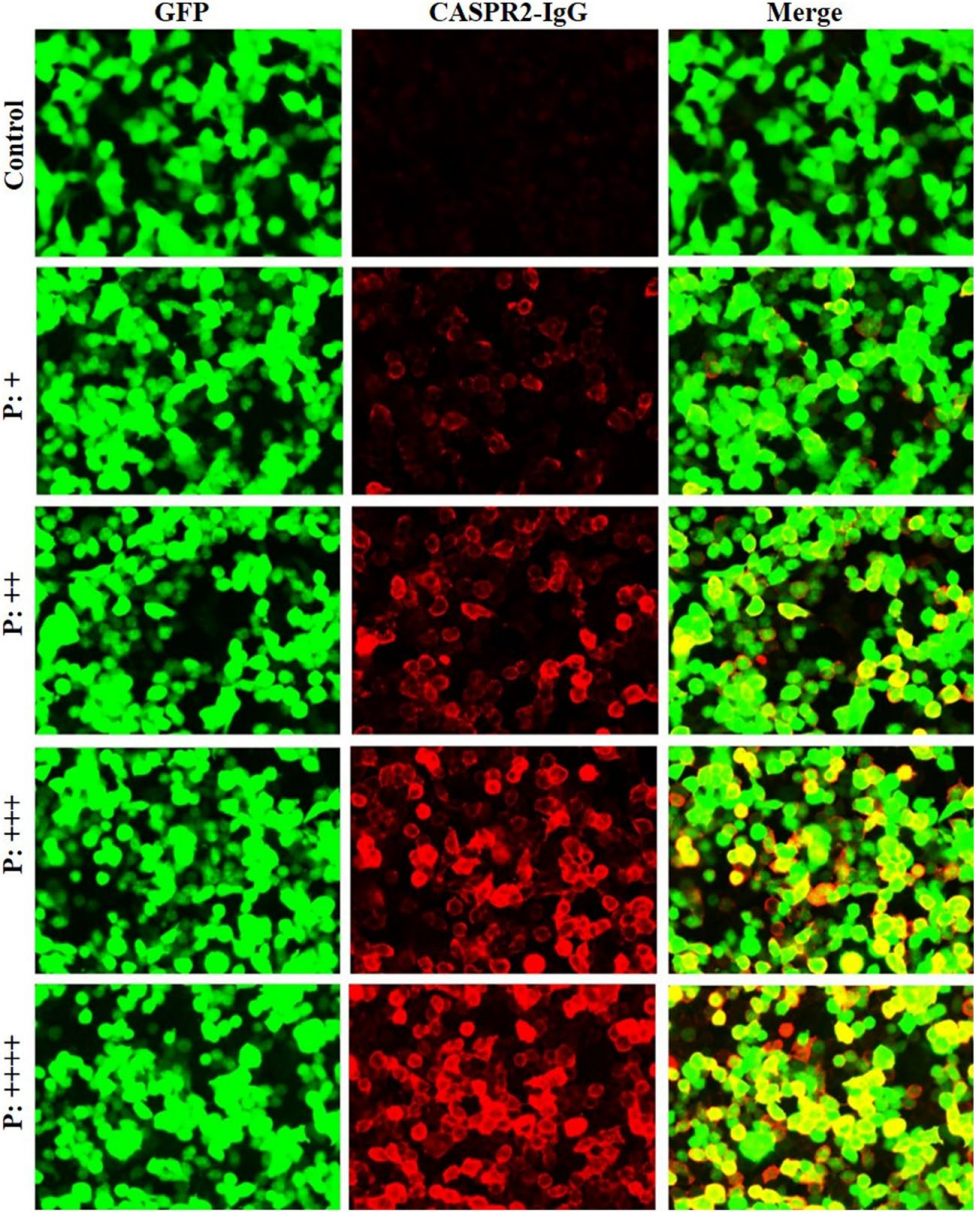
CASPR2-IgG in serum tested by a cell-based assay (CBA) using HEK293T cells transiently cotransfected with full-length human CASPR2 and pcDNA3.1-EGFP (Scale bar: 20 µm). The patient’s IgG bound to CASPR2-transfected cells and showed red fluorescence as a positive control.

According to the antibody titer, we used the symbols + to ++++ to represent the results of positive antibodies. Representative pictures of each interval are shown in Fig. 1. Due to the limitation of the conditions in China (The testing fee is expensive and no insurance policy), the CBA method used in our cohort is not live cell-based assay, bu antibody titer is generally lower than that of live cells.t fixed assay. Therefore, our analysis result of antibody titer is a semi quantitative analysis, according to the strength of the fluorescence signal.

### Ethical approval and consent to participate

The studies involving human participants were reviewed and approved by the Medical Ethics Committee of Hunan Children’s Hospital. Written informed consent to participate in this study was provided by the participants’ legal guardian/next of kin.

## Results

### General information of CASPR2-IgG positive patients

Indications for antibody testing for the patients included clinically suspected autoimmune encephalitis (AE), demyelinating encephalopathy, and epilepsy of unknown cause with an Epilepsy and Encephalopathy (APE2: as a model to predict the detection of these Abs based on patients’ clinical presentation and initial neurologic evaluation) score ≥ 4^7^. Over a 7-year period, more than 3000 samples from pediatric patients with clinically suspected neurological autoimmunity were tested. The most common positive antibodies were against NMDAR (128/1358 in CSF), MOG (96/550 in serum) and GFAP (45/278 in serum). Three LGI1 antibody-positive patients (3/2658 in serum) were found among our samples. There was no GAD-65 antibody positive in our study. A total of 26 anti-CASPR2-positive patients (26/2658 in serum, and 3/2016 in CSF) were identified among those examine. The results of the antibody titers for anti-CASPR2 were as follows: +++ for 2 patients, ++ for 2 patients, and + for 22 patients; 25 patients showed serum positivity and 3 showed CSF positivity (including 2 patients with both serum and CSF positivity). Three patients were double-antibody positive for anti-NMDAR antibody (2 in CSF, 1 in both serum and CSF) and 1 patient was double-antibody positive for anti-GABABR antibody in CSF.

The oligoclonal bands were detected in 4 patients, and no positive results were obtained. All the patients had done tumor screen examinations, including chest X-ray or lung CT, color Doppler ultrasound or CT for thyroid, reproductive system, organs or lymph nodes in abdominal and pelvic cavity; and peripheral blood tumor antibody screening in partial patients. No cases with tumors beyond the central nervous system were found.

Among the 26 anti-CASPR2-positive patients, 17 were male and 9 were female. The age of onset ranged from 5 months to 14 years: > 5 months but < 4 years, 4 patients; ≥ 4 years but < 7 years, 6 patients; ≥ 7 years but < 10 years, 11 patients; ≥ 10 years but < 13 years, 4 patients; ≥ 13 years, 1 patient. The most common onset age was 4–9 years. The median follow-up period was 22 months (range, 3–62).

### Clinical spectrum analysis of CASPR2 autoantibody-related disease

The clinical features, radiological and electrophysiological findings, treatment strategies and prognoses are summarized in Table 1. The key clinical phenotypes included encephalitis phenotype (including autoimmune encephalitis/encephalopathy/cerebellitis), intractable epilepsy, psychobehavioral abnormalities and combined tumors. Considering the possibility of false positive anti-CASPR2 antibodies, we classified the patients according to their main clinical phenotype, auxiliary examination, treatment and prognosis.

**Table 1:** Clinical features, auxiliary examinations, diagnosis and treatment strategies, prognosis in pediatric patients seropositive for CASPR2-IgG.

Patient no. Sex/ age of onset	Summary clinical	EEG	CSF:WBC *10^6^/l;Pro g/l	MRI	Anti-CASPR2 and other antibodies	Clinical syndrome	Treatment	mRS at onset/last f/u	Follow-up time (months)	outcome
Mainly manifestated as autoimmune encephalitis/encephalopathy/cerebellitis										
1.M/3y	Fever, seizures, coma, weakness	Slow wave background	N	Cortical lesions in O/T/P lobes	+ /serum	Autoimmune encephalopathy	IVIG	5/0	56	Improved to normal
2.M/7y	Fever, neuropathic pain, ataxia, dysfunction with sleep, and consciousness, psychiatric symptoms	Slow wave background	N	N	+ + /serum	Autoimmune encephalitis	Symptomatic	4/0	40	Improved, back to school, emotional agitation
3.M/9y	Fever, dysfunction with consciousness and sleep, myotonia and autonomic symptoms	Slow wave background	WBC 214, Pro 0.593	Lesions in O/P cortex, thalamus, globus pallidus, hippocampus and cerebral peduncle	+ + + /serum, + /CSF, GABABR: + /CSF	Morvan syndrome	IVIG + IVMP + Prednisone + Rituximab	5/0	14	Improved to normal
4.M/9y	Seizures, slow response, sleep disturbance, psychiatric symptoms	Slow wave background, delta rhythm bursts	N	N	+ /serum	Autoimmune encephalitis	IVIG + Prednisone	5/0	24	Improved, back to school, memory loss
5.M/5 m	Fever, seizures, coma, weakness, sleep disturbance	Slow wave background, sharp waves in right F	N	Lesions in globus pallidus, corpus callosum and cerebral peduncle	+ /serum	Autoimmune encephalopathy	Symptomatic	5/1	34	Developmental retardation. He could walk and speak independently at 2 years and 6 months old
6.F/2y	Fever, seizures, unconsciousness, sleep and movement disorders, psychiatric symptoms	Slow wave background, sharp waves in left T	WBC 70	Cortical lesions in F/T/P/O lobes, hippocampus and thalamus	+ /serum, NMDAR: + + /CSF	Autoimmune encephalitis	IVIG + IVMP + Prednisone	5/0	23	Improved to normal
7.F/12y	Fever, psychiatric symptoms sleep and movement disorders, myotonia	Slow wave background	N	N	+ /serum	Morvan syndrome	IVIG + Prednisone	4/0	18	Improved to normal
8.F/5y	Fever, seizures, slow response	Slow wave background, delta rhythm bursts	N	Lesions in F/T/P/O lobes, and caudate nucleus	+ /serum	Autoimmune encephalopathy	IVIG	2/0	11	Improved to normal
9.M/6y	Fever, seizures, weakness	spikes in left F	N	Cortical and subcortical lesions in F/T lobes	+ /serum	Autoimmune encephalopathy	IVMP + IVIG + Prednisone	3/0	9	Improved to normal
10.M/6y	Fever, ataxia, slow response, progress to hemiplegia, irritability	Slow wave background	WBC 138	Lesion in thalamus, caudate nucleus and cerebral peduncle	+ /CSF, NMDAR: + /CSF	Autoimmune encephalitis	IVIG + IVMP + Prednisone + Rituximab	4/1	4	Hemiplegia slightly
11.F/12y	Fever, headache, seizures, unconsciousness, psychological symptoms	Slow wave background	Pro 0.67	Lesion in bilateral thalamus	+ /serum	Autoimmune encephalitis secondary to Japanese encephalitis	IVIG + IVMP + Prednisone	5/0	14	Improved to normal
12.F/14y	Ataxia gait	N	WBC 59 Pro 0.72	N	+ /serum	Autoimmune cerebellitis	IVIG + IVMP + Prednisone	3/0	11	Improved to normal
13.M/9y	Abnormal mental and behavior, seizures, unconsciousness	Slow wave background, Spikes or sharp slow wave in left F/T area, EPC	N	Multiple lesions in white matter	+ /serum, NMDAR: + + + /CSF and serum	Autoimmune encephalitis	IVIG + IVMP + Prednisone + PE	5/0	4	Improved to normal
14.F/7y	Fever, headache, slow response	Slow wave background	WBC 390 Pro 0.6	Cortex edema, Lesion in bilateral thalamus and right T lobe	+ + /serum, + /CSF	Autoimmune encephalitis	IVIG + IVMP + Prednisone	2/0	11	Improved to normal
15.F/2y	Seizures, ataxia, irritability	Slow wave background	N	N	+ + + /serum	Autoimmune encephalitis	IVIG	3/0	23	Improved to normal
Mainly manifestated as refractory epilepsy:										
16.M/9y	Recurrent focal seizures	Right temporal spikes and sharp waves	N	Multiple cortical lesions	+ /serum	Refractory epilepsy; TSC	IVIG + AEDs	1/1	49	Epilepsy, seizure frequency decreased
17.M/6y	Recurrent focal seizures, neuropathic pain, irascibility	Slow wave background, delta rhythm bursts	N	N	+ /serum	Refractory epilepsy	IVIG + Prednisone + AEDs	2/1	28	Epilepsy, seizure frequency decreased
18.F/5y	Recurrent focal seizures	Left frontal sharp waves	NA	N	+ /serum	Refractory epilepsy	IVMP + Prednisone + AEDs	2/1	40	Epilepsy, seizure frequency decreased
19. M/7y	Recurrent focal seizures, EPC	Slow wave background, spikes in right frontal area, EPC	N	N	+ /serum	Refractory epilepsy	AEDs, IV Midazolam	2/0	21	Seizure free
20. M/8y	Recurrent focal seizures	Spikes or sharp slow wave in left P/T/O area	N	N	+ /serum	Refractory epilepsy	AEDS	1/1	62	Epilepsy, seizure frequency decreased
21.M/9y	Recurrent focal seizures	Spikes or sharp slow wave in left P/T area	N	Cortical lesions in left P area	+ /serum	Refractory epilepsy, FCD	IVMP + Prednisone + AEDS + lesion excision	2/0	24	Seizure free after lesion excision
Mainly manifestated as psychobehavioral abnormalities:20										
22.M/10y	Behavioral and psychological symptoms	N	N	N	+ /serum	Psychobehavioral abnormalities	IVIG + Prednisone	3/0	20	Improved to normal
23.M/5y	Behavioral and psychological symptoms	N	NA	N	+ /serum	Psychobehavioral abnormalities	Symptomatic	3/3	9	Abnormal mental and behavior
24.F/9y	Personality change and sleep disorder	N	NA	N	+ /serum	Psychobehavioral abnormalities	Symptomatic	1/0	16	Improved to normal
25.M/8y	Behavioral and psychological symptoms	N	N	N	+ + /serum	Psychobehavioral abnormalities	IVIG + IVMP + Prednisone + Mycophenolate mofeil	3/3	9	Abnormal mental and behavior
Accompanied with tumor:										
26.F/12y	Hemiplegia, headache	Low voltage in right C/P/T	N	Lesions in right globus pallidus and midbrain	+ /serum	Germinoma	IVMP + Prednisone	3/3	3	Hemiplegia

*M*: male, *F*: Female, *N*: normal, *F*: Frontal, P: Parietal, *O*: Occipital, *T*: Temporal, *AED*: antiepileptic drug, *CSF*: Cerebrospinal fluid, *WBC*: white blood cell in CSF, *Pro*: protein in CSF, *EEG*: electroencephalography, *MRI*: magnetic resonance imaging, *mRS*: modified Rankin Scale, *IVIG*: intravenous immunoglobulin, *IVMP*: intravenous methylprednisolone, *TSC*: tuberous sclerosis, *NA*: not available, *PE*: plasma exchange, *EPC*: epilepsia partialis continua, *FCD*: focal cortical dysplasia.

When other causes are excluded, the diagnostic criteria of autoimmune encephalopathy are: changes in mental state, including somnolence, restlessness, personality changes, and abnormal behavior; Symptoms persist for more than 24 h. Diagnostic criteria for AE: on the basis of encephalopathy, at least 2 of the following 6 items should be met: fever, seizures, focal neurological deficits, increased CSF cells (≥ 5/ul), new brain parenchyma abnormalities can be seen on brain imaging, which is consistent with EEG abnormalities of encephalitis^2,8^. Sometimes, the symptoms and characteristics of encephalitis, encephalopathy, cerebellitis and other diseases overlap each other, making it difficult to distinguish them. Therefore, we will discuss these diseases together. However, according to the Cellucci criteria for pediatric AE in 2020^9^, patients N2, N4, N7 and N15 could only be diagnosed as possible AE because of the lack of paraclinical evidence of nouroinflammation. Considering that these patients possessed the evidence of acute or subacute onset, more than 2 features of neurologic dysfunctions, presence of antibodies in serum and/or CSF, and exclusion of other etiologies. We still classified them into CASPR2 antibody-related disease.

### CASPR2 autoantibody-related disease mainly manifested as encephalitis phenotype in 15 patients

Fifteen patients presented with encephalitis phenotype: 10 with autoimmune encephalitis (2 of whom presented with Morvan syndrome), 4 with typical clinical features of autoimmune encephalopathy, and 1 with autoimmune cerebellitis. Three patients presented with AE coexisting with anti-NMDAR antibody positivity. One patient presented with Morvan syndrome coexisting with anti-GABABR antibody positivity. One patient presented with autoimmune encephalitis secondary to Japanese encephalitis. However, based on Cellucci criteria^9^, patient N6, N10 and N13 could be diagnosed as anti-NMDAR encephalitis, patient N3 could be diagnosed as anti-GABABR encephalitis, anti-CASPR2-positive in these patients may be just a coexistence phenomenon.

The results of the antibody titers for anti-CASPR2 were as follows: +++ for 2 patients, ++ for 2 patients, and + for 11 patients; 3 patients showed CSF positivity (including 2 with both serum and CSF positivity).

The age of onset ranged from 5 months to 14 years, the median onset age was 6 years and 5 months. The most common symptoms of those 15 patients included disorders of consciousness, fever, psychological symptoms/abnormal behaviors, sleep disorders, seizures, movement disorder, autonomic symptoms, peripheral nerve hyperexcitability/neuromyotonia and weakness/hemiplegia. The autonomic symptoms included 5 cases of hyperhidrosis, 4 cases of hypertension, 4 cases of gastrointestinal dysfunction and 2 cases of paroxysmal arrhythmia. The symptoms such as hyperhidrosis, irritability and tachycardia caused by high-dose of steroid administration were not included. There was no patient with simple visual impairment or paraplegia.

**Figure 2:**
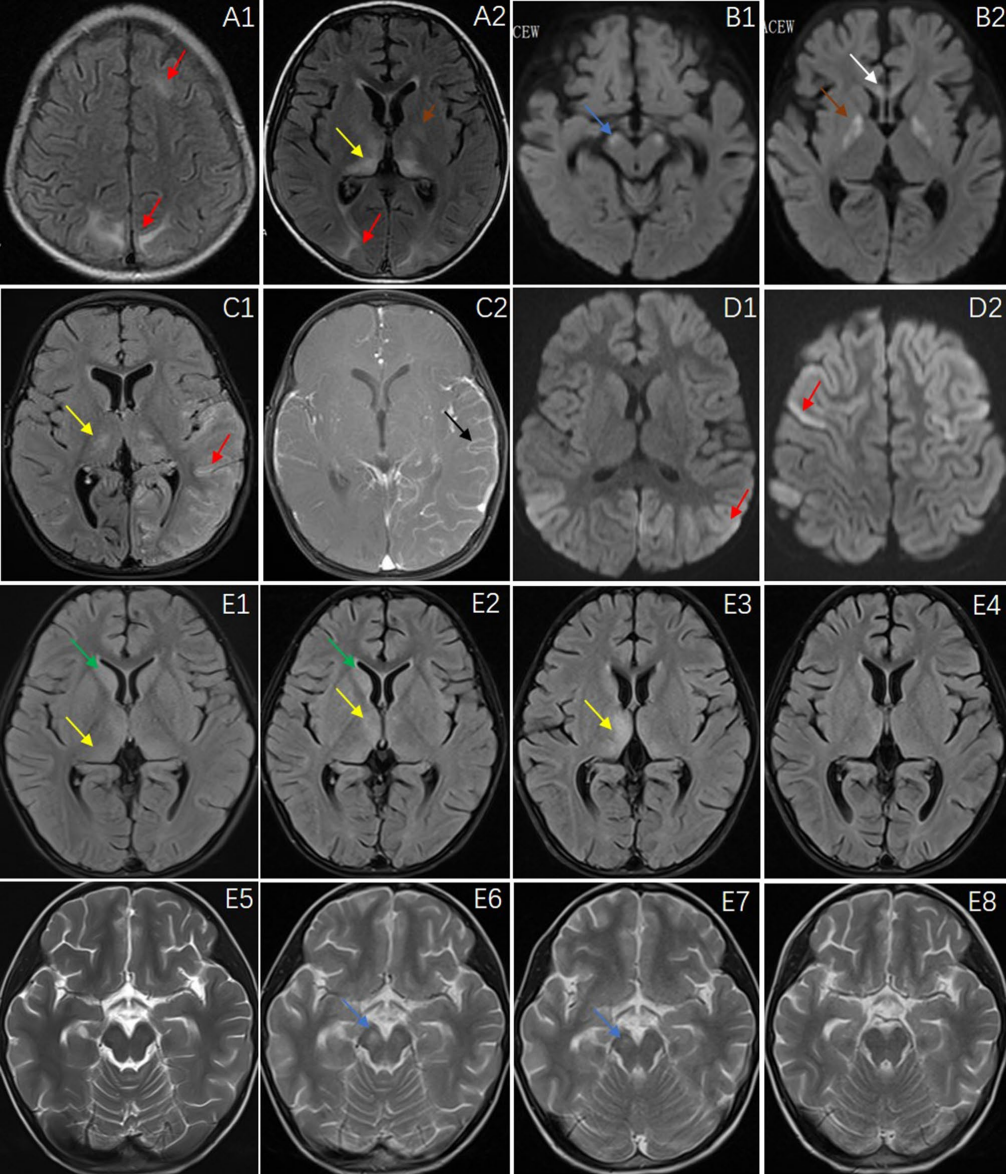
Brain MRI features of patients N.3, N.5, N.6, N.8 and N.10. Patient N.3, a 6-year-old boy, had a clinical-radiological presentation of Morvan syndrome including multifocal lesions in the occipital and parietal cortex and thalamus (**A1**, **A2**). Patient N.5, a 5-month-old boy, presented with encephalopathy and bilateral MRI abnormalities in the globus pallidus, corpus callosum and cerebral peduncle (**B1**, **B2**). Patient N.6, a 2-year-old girl, had a clinical-radiological presentation of autoimmune encephalitis, including cortical lesions in the frontal, parietal, occipital and temporal lobes and thalamus and cortical lesions showing enhancement with gadolinium administration (**C1**, **C2**). Patient N.8, a 5-year-old girl, presented with two episodes of fever and seizures within a week. Brain MRI showed extensive cortical lesions in the frontal, parietal, occipital and temporal lobes (**D1**, **D2**). Patient N.10, a 6-year-old boy, presented with fever, ataxia, slow responses. Brain MRI (**E1**–**E8**) showed a series of changes including lesions in the thalamus, caudate nucleus and cerebral peduncle (E1/E5, E2/E6, E3/E7, E4/E8, representing D4, D16, D30, and D50 after onset, respectively). Fluid-attenuated inversion recovery sequences were used in (**A1**, **A2**, **C1**, **E1**–**E4**, and **C2**, which show enhancement with gadolinium administration. T2 sequences were used in (**E5**–**E8**). Diffusion weighted imaging was used in (**B1**, **B2**, **D1** and **D2**). Red arrows represent multiple cortical lesions, yellow arrows: thalamus, blue arrows: cerebral peduncle, green arrows: caudate nucleus, brown arrows: globus pallidus, white arrow: corpus callosum, black arrow: meningeal enhancement.

**Figure 3:**
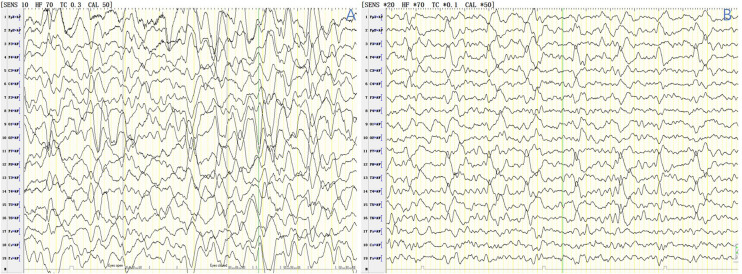
EEG features of patient N4, boy, aged 6 years, who presented with refractory epilepsy. (**A**) diffuse delta rhythm during awake period; (**B**) periodic waves from right frontal and temporal area during sleep.

Brain MRI (Fig. 2) revealed abnormalities in 10 patients (66.7%). The most common sites of lesions were the cerebral cortex (widely distributed across the frontal, parietal, occipital and temporal lobes), thalamus, caudate nucleus, cerebral peduncle, white matter, hippocampus, globus pallidus and corpus callosum. Electroencephalography (EEG) recordings revealed a slow wave background in 13 patients (86.7%), 2 (21.43%) of whom had bursts of delta rhythm (Fig. 3). Epileptiform discharges, originating from the frontal and temporal lobes, were found in 4 patients (26.7%), and one patient manifested with epilepsia partialis continua (EPC). All 15 patients underwent lumbar puncture examinations, 5 showed elevated WBC in CSF, and 4 showed elevated protein in CSF. Changes in the CSF were not very common in CASPR2-related patients. The CSF pathogen tests were negative in all patients.

For the treatment strategies, 13 patients received immunotherapy, including intravenous immunoglobulin (IVIG), IVIG plus prednisone, IVIG plus intravenous methylprednisolone (IVMP) plus prednisolone, IVMP plus prednisone, IVIG plus IVMP plus prednisone plus rituximab, and IVIG plus IVMP plus prednisone plus plasma exchange (PE). The modified Rankin Scale (mRS) score was used to evaluate the efficacy and prognosis. After treatment, twelve patients had an mRS score of 0, and one of them had memory loss. One patient had an mRS score of 1, with fine motor disorder of left hand; the follow-up time of the patient was only 3 months after immunotherapy, and the patient is in recovery. Two patients received symptomatic therapy (for economic reasons and given the side effects of hormones, the family refused immunotherapy). The mRS scores before treatment were 4 and 5. After 3 years of follow-up, the symptoms of these 2 patients improved gradually, and the mRS scores were 1 and 0, respectively; one patient had emotional agitation, and the other had developmental delay.

### Brief case descriptions

Each patient's clinical features and treatment process have their own characteristics. Here, we show several interesting cases separately.

Patient N.3, a boy aged 6 years, developed neuromyotonia and neuropathic pain, movement disorders, sleep disorders, irritability, eating difficulties and cognitive changes. Both CASPR2 and GABABR antibodies were detected in his serum and CSF. He was treated with intravenous immune globulin (IVIG) (2 g/kg/day for 5 days) and IV methylprednisolone (IVMP) (3 cycles of 20 mg/kg/day for 3 days alternating with 4 days of rest). One month later, his symptoms had not been relieved, his CASPR2 titers had not decreased, and brain MRI (Fig. 2-A1/A2) revealed new lesions. He was treated with rituximab (375 mg/m^2^, once a week, 4 times) and improved remarkably. One month after rituximab treatment, all his symptoms were relieved, his MRI results had become normal, and the CASPR2 antibody test was negative. Moreover, during the treatment, we found that carbamazepine had a significant effect on relieving the symptoms of myotonia.

Patient N.5 was the youngest patient, only 5 months old at symptom onset. His main symptoms included fever, recurrent seizures, disturbance of consciousness, and irritability. Brain MRI (Fig. 2-B1/B2) showed lesions in the globus pallidus, corpus callosum and cerebral peduncle. The WBC, protein, bacterial culture and macrogene examination for virus nucleic acid in CSF were normal. CASPR2 antibody positive was found in the serum. He received symptomatic treatment, including assisted ventilation. His symptoms gradually improved, and his development gradually progressed after rehabilitation training. At the age of 2 years and 6 months, he could speak and walk independently.

Patient N.6, a girl aged 2 years, developed fever, recurrent seizures, unconsciousness, sleep and movement disorders, and psychological symptoms. Both CASPR2 and NMDAR antibodies were detected. Brain MRI (Fig. 2-C1/C2) showed extensive lesions in the cortex and thalamus, with meningeal enhancement of the cortical lesions. The symptoms in the acute stage were very severe but improved greatly after IVIG and half a year of steroid treatment. The patient was able to attend school normally and had normal intelligence.

Patient N.8, a girl aged 5 years, presented with two episodes of fever and seizures within a week. The symptoms were resolved without treatment. Brain MRI showed extensive cortical lesions (Fig. 2-D1/D2). However, we still used IVIG to prevent aggravation of the clinical symptoms. During the 15-month follow-up, no symptom recurrence was observed.

Patient N.10, a boy aged 6 years, was admitted to the hospital because of fever, ataxia, and slow responses. EEG showed a low wave background, and brain MRI showed lesions in the thalamus and caudate nucleus (Fig. 2-E1/E5), with elevated WBCs in the CSF. Anti-CASPR2 antibody was positive in the CSF and negative in the serum, while NMDAR was initially negative in both the CSF and serum. He was treated with IVIG+IVMP, and the clinical symptoms were improved. One month after he was discharged from the hospital, he presented with hemiplegia and irritability. Brain MRI revealed new lesions in the cerebral peduncle (Fig. 2-E2/E6), and anti-NMDAR antibody was positive in the CSF, but anti-CASPR2 antibody was negative. He was treated with IVMP again and then rituximab. The lesions on brain MRI (Fig. 2; E3/E4/E7/E8, E1/E5, E2/E6, E3/E7, and E4/E8 represent D4, D16, D30, and D50 after onset, respectively) were decreased after immunotherapy. The hemiplegia improved (at 1 month after discharge) but incompletely, and the patient remains in follow-up.

Patient N.11, a 12-year-old girl, was initially diagnosed with Japanese encephalitis. On 24, July, 2021, she presented high fever, convulsions, disturbance of consciousness and central respiratory failure. Brain MRI showed symmetrical lesions of bilateral thalamus, elevated WBC and positive antibody to Japanese encephalitis in CSF in acute stage. After symptomatic treatment, her clinical symptoms gradually relieved and she was discharged home on August 19. On August 24, she developed convulsions, psychological symptoms and disturbance of consciousness again. Brain MRI shown lesions in the bilateral thalamus. CASPR2 antibody was positive, while NMDAR and other immune antibodies were negative. She was considered to have CASPR2 antibody-related autoimmune encephalitis secondary to encephalitis B, and the symptoms were cured after immunotherapy.

Patient N.12, a 14-year-old girl, mainly presented with an ataxic gait, elevated WBC and protein in the CSF, without symptoms of encephalitis or encephalopathy. Her EEG and brain MRI were normal. She was diagnosed with autoimmune cerebellitis and recovered to normal after administration of IVIG+IVMP, and oral prednisone for 1 month. She was the only patient with a normal EEG among 15 patients diagnosed with CASPR2-related autoimmune diseases.

### CASPR2 autoantibody-related disease mainly manifested as refractory epilepsy in 6 patients

Six patients presented with refractory epilepsy, which manifested as recurrent focal seizures. The antibody titers for anti-CASPR2 were + in serum for all patients. Brain MRI revealed abnormalities in one patient with tuberous sclerosis (TSC) and another with focal cortical dysplasia (FCD), while the remaining 4 patients had normal MRIs. EEG results were abnormal in all patients: epileptic charges were observed in 5 (83.3%) patients, a slow wave background in 2 (33.3%) patients, bursts of delta rhythm in one patient, and epilepsia partialis continua (EPC) in one patient. WBC, protein concentration and autoimmune antibodies in CSF were examined in four patients and were normal.

Patient N.16 presented with focal seizures. Brain MRI revealed multiple cortical lesions. He was treated with IVIG and antiepileptic drugs (AEDs), but recurrent seizures persisted. Genetic tests showed TSC1 gene pathogenic mutations, which supported a diagnosis of TSC.

Patient N.17 had neuropathic pain and irascibility, with no specific changes on MRI and CSF examination. He was treated with IVIG plus prednisolone and AEDs, but recurrent seizure attacks persisted during the 3-year follow-up.

N.21 also presented with recurrent focal seizures, and EEG and MRI indicated left parietal abnormalities. He was treated with IVMP plus prednisone and AEDs but responded poorly. Finally, he achieved a seizure-free status after surgery, and pathological examination confirmed a diagnosis of FCD.

For the treatment strategies, all patients were treated with antiepileptic drugs (AEDs); 1 patient was treated with IVIG, one with IVIG plus prednisolone, and one with IVMP plus prednisolone. The mRS scores before treatment were 2 in four patients and 1 in two patients. After treatment, the mRS scores were 1 in four patients. One patient achieved a seizure-free status after AED treatment. The FCD patient was seizure free after surgery. The seizure frequency of the other 4 patients decreased. Among these 6 patients, the use of immunotherapy had little correlation with prognosis.

### CASPR2 autoantibody-related disease mainly manifested as psychobehavioral abnormalities in 4 patients

Four patients presented with psychobehavioral abnormalities. Three patients mainly manifested with self-talking, giggling, hallucinations, irritability, social disorders, fear, etc. One patient manifested with personality changes and sleep disorders. The results of the eCASPR2 antibody titers were all positive in serum: ++ for 1 patient and + for 3 patients. Brain MRI and EEG were normal in all patients. WBC, protein concentration and autoimmune antibodies in CSF were examined in two patients, and both were normal.

Patient N.22 was treated with IVIG plus prednisolone. His symptoms improved, with pre-/post-treatment mRS scores of 3/0. Patients N.23 and N.24 were only given symptomatic treatment; N.24 improved to normal, with mRS scores before/after treatment of 1/0, while the clinical symptoms of N.23 did not improve, with mRS scores before/after treatment of 3/3. Patient N.25 (with antibody titer ++) was treated with IVIG+IVMP + prednisone + mycophenolate mofeil, but the clinical symptoms did not improve; the mRS scores before/after treatment were 3/3.

### CASPR2 autoantibody-related disease accompanied with tumor in 1 patient

Patient N.26 manifested with hemiplegia of the left limb and headache. Brain MRI showed lesions in the right globus pallidus and midbrain. EEG showed low voltages in the right central, parietal and temporal areas, with normal WBC and protein concentrations in the CSF. The CASPR2 antibody titer was + in serum. He was treated with IVMP+prednisone but showed no improvement. Finally, he was pathologically diagnosed with germinoma by brain biopsy.

### Comprehensive analysis of patients false positive for anti-CASPR2 neurological autoimmunity

Based on the clinical symptoms, MRI, EEG, CSF changes, antibody titers, copositivity with other antibodies, response to immunotherapy and other significant findings (such as past history and pathological examinations), we conducted a comprehensive analysis of the 26 patients (Table 2) to distinguish between true- and false-positive cases of CASPR2-related autoimmunity and to explore the correlation between the clinical phenotype and positive anti-CASPR2 antibody.

**Table 2:** The Summary of clinical symptoms, auxiliary examination and treatment response between different phenotypes of the patients seropositive for CASPR2-IgG.

	Autoimmune encephalitis/encephalopathy/cerebellitis	Refractory epilepsy	Psychobehavioral abnormalities	Accompanied with tumor
Number	15	6	4	1
M:F	8:7	5:1	3:1	1:0
Symptoms				
Disorders of consciousness	10	0	0	0
Fever	8	0	0	0
Psychological symptoms/ abnormal behavior	8	1	4	0
Sleep disorders	8	0	2	0
Seizures	7	6	0	0
Movement disorder	5	0	0	0
Autonomic symptoms	5	0	0	0
Peripheral nerve hyperexcitability/neuromyotonia	5	1	0	0
Weakness/ hemiplegia	4	0	0	1
Elevated WBC/protein in CSF	6	0	0	0
MRI changes				
Cortical lesions	6	2	0	0
Thalamus	5	0	0	0
Caudate nucleus	3	0	0	0
Cerebral peduncle	3	0	0	1
White matter	2	0	0	0
Hippocampus	2	0	0	0
Globus pallidus	2	0	0	1
Corpus callosum	1	0	0	0
EEG				
Slow wave background	13	2	0	0
Epileptiform discharges	4	5	0	0
Higher antibody titers	4	0	0	0
Co-positive with other antibodies	4	0	0	0
Good response to immunotherapy	13/13	1/4	1/2	0/1
Other situations	1 case was secondary to Japanese encephalitis	FCD: 1 case; TSC: 1 case	–	Germinoma

*M*: male, *F*: female, *CSF*: cerebrospinal fluid, *WBC*: white blood cell, *EEG*: electroencephalography, *MRI*: magnetic resonance imaging, *TSC*: tuberous sclerosis, FCD: focal cortical dysplasia.

Among the 26 anti-CASPR2-positive patients, the 15 patients who presented with autoimmune encephalitis/encephalopathy/cerebellitis could probably be diagnosed with anti-CASPR2 neurological autoimmunity, as their clinical symptoms and signs and EEG, CSF and MRI changes were consistent with the characteristics of autoimmune diseases. The anti-CASPR2 antibody titers were higher and more frequently accompanied by other autoimmune antibodies than those of the other patients. The responses to immunotherapy were very well.

For the 6 patients who manifested with refractory epilepsy, we could not confirm that their etiology was related to the CASPR2 antibody. The core symptoms of these patients were mainly recurrent epilepsy. Patient N.17 was accompanied by emotional changes such as irritability, but the effect of immunotherapy was poor; the CSF and MRI results showed no specific changes, and the antibody titers were low. The use of immunotherapy had little effect on prognosis.

For the 4 patients who manifested with psychobehavioral abnormalities, we cannot confirm that their etiology was related to the CASPR2 antibody. They showed no solid evidence of encephalopathy and encephalitis except for psychobehavioral abnormalities and no specific changes in the CSF and on MRI. The use of immunotherapy had little effect on prognosis.

Obviously, in the patient diagnosed with germinoma, the positive CASPR2 antibody was likely to be related to the tumor, and immunotherapy was not effective. This suggests that the CASPR2 antibody positivity in this patient may be a paraneoplastic syndrome.

## Discussion

The continual discovery of novel forms of encephalitis associated with autoimmune antibodies has changed the paradigms for diagnosing and treating disorders that were previously unknown or mischaracterized^10^. To date, anti-NMDAR encephalitis has been identified as the most common subtype in pediatric patients, but reports of anti-MOG antibody disease are increasingly frequent. In this study, we reported 26 children with positive anti-CASPR2 antibody, to analysis their clinical features, MRI changes, treatment strategies and outcomes, aim to provide clues for clinicians to understand CASPR2 autoimmunity in children.

Anti-CASPR2-related encephalitis in children is rarely reported. Only 0.1% of 13,319 pediatric patients undergoing serological evaluation were identified as positive for LGI1 and CASPR2-IgG^11^. In a review of LGI1 and CASPR2 autoimmunity in children, 37 pediatric patients with CASPR2 and/or LGI1 autoimmunity were identified in the literature: 37.8% of patients had LGI1 antibodies, 37.8%had CASPR2 antibodies, and 24.3% of patients were double positive for LGI1 and CASPR2 antibodies^12^. In our study, we tested for NMDAR-IgG, AMPA1-IgG, AMPA2-IgG, LGI1-IgG, CASPR2-IgG, GABABR-IgG, MOG-IgG, GFAP-IgG, and AQP4-IgG in patients with clinically suspected neurological autoimmunity; NMDAR-IgG and MOG-IgG positivity were most common. The positive rate for CASPR2 was lower than that for NMDAR and MOG but was not very low overall. In the last year, as understanding of the disease has increased, we have been able to identify more than 10 positive patients. Previous literature has suggested that the rarity may be related to the fact that this antibody is not used as part of a routine examination^13^. In total, we screened 26 positive patients out of approximately 3,000 patients. In contrast to NMDARs, which were mainly positive in the CSF, the positive rate for CASPR2 was markedly higher in serum (25/26 vs 3/26 for CSF). The phenomenon of co-positive with other antibodies were common. Anti- CASPR2 co-positive with pathogenic antibodies of anti-NMDAR and anti- GABABR suggested that CASPR2 antibody maybe only reflect an immune activation phenomenon, and it is not necessarily a pathogenic antibody itself. For those patients had positive CSF antibodies, they tended to behave an ‘adult-like’ CASPR2 phenotype and have co-positive antibodies. However, we cannot confirm which is the responsible antibody or pathogenic antibody for these diseases. Comprehensive analysis may be required according to antibody titer, clinical symptoms and phenotype, and treatment response. Perhaps, anti-CASPR2-positive only reflects a state of immune disorders in these diseases, and the underlying mechanism remains to be further studied.

False positivity for CASPR2 antibody has been identified in the clinic^13^, and so we, too, considered the possibility of false positives. For those enrolled 26 patients, the diagnosis was confirmed by a comprehensive analysis of multiple factors, such as clinical manifestations, auxiliary examinations, antibody titers and responses to immunotherapy treatment (remission of symptoms, improvement on brain imaging, decrease in or negative conversion of antibody titers). We were unable to rule out the possibility of false positives in 11 patients (6 who manifested with refractory epilepsy, 4 who manifested with psychobehavioral abnormalities, and 1 diagnosed with germinoma) among the CASPR2 antibody-positive patients. The false positive rate was very high (42.3%, 11/26), mainly due to patients with low antibody titers and a lack of typical encephalitis/encephalopathy symptoms or corresponding changes on EEG and brain MRI or in the CSF. However, CASPR2 antibody positivity was very frequent in patients with refractory epilepsy and psychobehavioral abnormalities, some of whom seemed to respond positively to immunotherapy. Tan et al. reported 6 children with CASPR2 autoimmunity, Psychotic disorder was the most common symptom^14^. In clinic, there were many autoimmune diseases, such as MOG antibody and NMDAR antibody mediated autoimmune encephalitis, presented isolated seizures at the onset stage, then proceed to appearing typical encephalitis manifestations in several months or even years^15,16^. Therefore, for these cases we reported, the relationship between CASPR2 antibody and intractable seizures or psychobehavioral abnormalities needs to be further confirmed with larger samples and longer follow-up time.

The most commonly reported presenting symptoms in adults include psychiatric symptoms, seizures, cognitive disturbance/memory impairment, sleep disorders, autonomic disorders and peripheral nerve hyperexcitability/neuromyotonia^17^. Among the 15 patients considered to have CASPR2-related autoimmunity in this study, the most common symptoms were similar to those in adults, but also included fever. Considered fever may be related to the history of prodromal viral infection and could further induce immune disorders, leading to immune encephalitis. Among our patients, autoimmune encephalitis/encephalopathy was the most common phenotype; among them, only 2 patients manifested with Morvan syndrome, and there was one patient only manifested with cerebellar ataxia. This difference from adults may be a specific characteristic of the disease in pediatric patients. Also, this maybe suggest anti-CASPR2 positive merely is coexistence of AE caused by other pathogenic antibodies, when we see positive low titer of anti-CASPR2 positive in serum, we can consider testing other neuronal autoantibodies for the final diagnosis.

Anti-NMDAR encephalitis can be secondary to herpes simplex virus encephalitis, Japanese encephalitis or other forms of viral encephalitis^5^. In our study, we also found positive CASPR2 antibody in patients with immune encephalitis secondary to Japanese encephalitis. This is the first report of CASPR2-associated immune encephalitis secondary to viral encephalitis. Carreno et al. reported that CASPR2, GAD, Ma2, LG11 antibodies positive in drug resistant temporal lobe epilepsy^18^. Additionally, we found CASPR2 antibody positivity in TSC, FCD and germinoma patients, phenomena that have not been reported in previous studies. Whether there is a correlation between them and whether immune factors are involved in the pathogenesis of these diseases is worthy of in-depth study. Perhaps, in these cases, anti-CASPR2 positive only reflects that blood brain barrier is disrupted by other things. Combine the contents discussed above, we need consider the pathogenicity of CASPR2 in all AE patients, especially when the titer is very low and there is insufficient evidence of neuroinflammation.

The auxiliary examination of CASPR2-related diseases has low specificity. Among our patients, elevated leukocytes or protein in CSF was observed in only 6. Also, in a review published in 2020, the abnormality of elevated WBC in CSF was 52/180 (28.9%)^4^. A slow wave background was the most common EEG finding, observed in the 14 patients who presented with autoimmune encephalitis/encephalopathy, 3 of whom had delta rhythms. Only one patient, who manifested with autoimmune cerebellitis, had normal EEG. EEG changes involving a slow wave background with a delta rhythms may thus be a diagnostic indicator of CASPR2 autoantibody disease.

Brain MRI is an important examination in the diagnosis of autoimmune encephalitis. Previous studies have observed abnormalities in over 50% of adult patients, with the most common sites of lesions in anti-CASPR2-related encephalitis being the temporal lobe and hippocampus^4^. We found that 66.7% of the patients had abnormalities on brain MRI. The cerebral cortex, including the frontal, parietal, occipital, and temporal lobes, was the most commonly involved lesion site, followed by the thalamus. In addition to the cortex and thalamus, other parts of the brain can be involved, including the basal ganglia, white matter, brain stem, and corpus callosum. Our study shows that the MRI features of CASPR2 autoantibody disease mainly involve lesions of the cerebral cortex, but there is no evidence to indicate which region of the cortex is most commonly involved.

According to the analysis of treatments and prognoses, for most CASPR2-related autoimmunity, immunotherapy strategies can improve the patients’ outcomes. this is consistent with the results in another study, Tan reported six patients, all patients had favorable outcomes with recurrence rate at 0%^14^. The intensity of immunotherapy differs depending on the clinical conditions of the patient. Most immunotherapy regimens consist of IVIG and/or steroids; however, two patients were treated with rituximab because IVIG plus IVMP did not lead to a full recovery. The youngest patient, 5 months of age, was treated only with IVIG in the acute stage, resulting in developmental delay. However, at the age of approximately 2.5 years, he was able to communicate with others and walk independently. Among the patients manifesting with refractory epilepsy and psychobehavioral abnormalities accompanied by tumors, some had a good response to immunotherapy, while others did not. We cannot confirm whether these diseases are related to immune disorders, and thus the efficacy of immunotherapy is uncertain.

Relapse of CASPR2-related autoimmunity was reported in adults. The reported relapse rate was between 16 and 37.5%^3,5^. The Mayo laboratory reported 6 children, two patients relapsed and required maintenance immunotherapy^6^. In our cohort, Patient N.10, who was diagnosed as autoimmune encephalitis firstly with anti-CASPR2 antibody positive in the CSF. He was treated with IVIG+IVMP, and the clinical symptoms were improved. One month later, he was relapse and brain MRI revealed new lesions with anti-NMDAR antibody positive in the CSF, but anti-CASPR2 antibody was negative. No other patients relapse. Relapse in children are rarely reported, may be due to the small sample size and short follow-up time.

In conclusion, CASPR2 autoantibody disease is not very rare in children and can occur in infancy. But this type of encephalitis occurs more frequently in older children. The most common symptoms include disorders of consciousness, fever, psychological symptoms/abnormal behavior, sleep disorders, seizures, movement disorders, autonomic symptoms, peripheral nerve hyperexcitability/neuromyotonia and weakness/hemiplegia. The most commonly involved part of the brain on MRI examination is the cerebral cortex. EEG changes involving a slow wave background, high antibody titers, co-expression with other antibodies, and well response to immunotherapy may help lead to an accurate diagnosis. The most common clinical phenotypes are encephalitis phenotype (including autoimmune encephalitis/encephalopathy/cerebellitis). We also identified patients with CASPR2-related immune encephalitis secondary to Japanese encephalitis and autoimmune encephalitis combined with other antibodies. In addition, we found anti-CASPR2 antibody positivity in many patients manifested as refractory epilepsy and unexplained psychobehavioral abnormalities. These patients have atypical encephalitis symptoms and lack obvious imaging and CSF changes, and the efficacy of immunotherapy remains unconfirmed. The false positive rate is very high, but we still cannot completely rule out whether immune factors are involved in the pathogenesis of the disease in these patients; thus, long-term follow-up and large sample research are needed. We also found anti-CASPR2 antibody positivity in patients with TSC, FCD and germinoma, all of which suggest that CASPR2 antibody positivity is nonspecific. The overall prognosis of these patients is good. Rituximab can be used in patients with a poor response to conventional immunotherapy.

## Data Availability

The datasets used and/or analyzed during the present study are available from the corresponding author on reasonable request.

## Abbreviations

AE: Autoimmune encephalitis
CASPR2: Contactin-associated protein-like 2
CSF: Cerebrospinal fluid
NMDAR: N-methyl-d-aspartate receptor
GABABR: γ-Aminobutyric acid B receptor
MRI: Magnetic resonance imaging
EEG: Electroencephalography
VGKC: Voltage-gated potassium channel
AMPA: Anti-α-amino-3-hydroxy-5-methyl-4-isoxazolepropionic acid
LGI1: Leucine-rich glioma inactivated 1
GAD65: Glutamic acid decarboxylase 65
MOG: Myelin oligodendrocyte glycoprotein
GFAP: Glial fifibrillary acidic protein
AQP4: Aquaporin-4
Ma2: Melbourne assessment 2
APE2: Antibody prevalence in epilepsy and encephalopathy
CBA: Cell-based assay
PBS: Phosphate-buffered saline
EPC: Epilepsia partialis continua
WBC: White blood cell
AED: Antiepileptic drug
Pro: Protein
mRS: Modified Rankin Scale
IVIG: Intravenous immunoglobulin
IVMP: Intravenous methylprednisolone
TSC: Tuberous sclerosis
NA: Not available
PE: Plasma exchange
FCD: Focal cortical dysplasia
